# Risk and protective factors associated with health-related quality of life in children and adolescents with ADHD in Germany – Findings from the consortium project INTEGRATE-ADHD

**DOI:** 10.25646/12315

**Published:** 2024-09-18

**Authors:** Martha Gilbert, Ulrike Ravens-Sieberer, Robert Schlack, Ann-Kristin Beyer, Marcel Romanos, Thomas Jans, Julian Witte, Peter Heuschmann, Cordula Riederer, the INTEGRATE-ADHD Study Group, Anne Kaman

**Affiliations:** 1 University Medical Centre Hamburg-Eppendorf, Department of Child and Adolescent Psychiatry, Psychotherapy and Psychosomatics, Research Section ‘Child Public Health’, Hamburg, Germany; 2 Robert Koch Institute, Department of Epidemiology and Health Monitoring, Berlin, Germany; 3 University Hospital Würzburg, Centre of Mental Health, Department of Child and Adolescent Psychiatry, Psychosomatics and Psychotherapy, Würzburg, Germany; 4 Vandage GmbH, Bielefeld, Germany; 5 University of Würzburg, Institute of Clinical Epidemiology and Biometry, Würzburg, Germany; 6 University Hospital Würzburg, Clinical Trial Centre, Würzburg, Germany; 7 University Hospital Würzburg, Institute for Medical Data Sciences, Würzburg, Germany; 8 DAK-Gesundheit, Hamburg, Germany

**Keywords:** ADHD, Health-related quality of life, Health care utilisation, Risk factors, Protective factors, Resources, Social support, Children, Adolescents, Parents, Mental health

## Abstract

**Background:**

The health-related quality of life (HRQoL) of individuals living with Attention-deficit/hyperactivity disorder (ADHD) is known to be impaired. Identifying factors that influence HRQoL can provide important information for the development of prevention and intervention programmes for affected children and adolescents. The aim of the present study was to investigate health care-related and psychosocial risk and protective factors for HRQoL in children and adolescents with an administrative ADHD diagnosis.

**Methods:**

In the consortium project INTEGRATE-ADHD, *n* = 4,809 parents of children and adolescents aged 7 to 17 years participated in an online survey between October 2021 and August 2022 and answered questions regarding HRQoL (KIDSCREEN-27), health care utilisation, and psychosocial risk and protective factors. Multiple linear regression analyses were conducted to assess the association between these factors and the five HRQoL dimensions of the KIDSCREEN-27.

**Results:**

Findings indicate that parental psychopathology and parental burden were risk factors for lower HRQoL in children and adolescents with ADHD. Further, a positive association was found between the five HRQoL dimensions and the psychosocial factors family climate and social support, indicating that these are protective factors.

**Conclusions:**

The results highlight the importance of prevention and intervention programmes for individuals with ADHD that consider parental mental health and aim to strengthen resources such as the availability of good family climate and social support.

## 1. Introduction

Attention-deficit/hyperactivity disorder (ADHD) is characterised by signs of developmentally inappropriate inattentiveness, impulsivity, and/or hyperactivity. According to DSM-5, the Diagnostic and Statistical Manual of Mental Disorders, and ICD-10, the International Statistical Classification of Diseases and Related Health Problems, these symptoms must occur over a period of at least six months and have a negative impact on academic, occupational, and social functioning [1, 2]. ADHD is regarded as one of the most prevalent neurodevelopmental disorders among children and adolescents worldwide. According to a meta-analysis of 135 studies, the global prevalence rate of ADHD in children and adolescents is about 5 % and has been found to be relatively stable over time [3]. Comparable prevalences were reported in a representative German sample of children and adolescents. While the overall prevalence in the age group of 3- to 17-year-olds was 4.4 %, boys were twice as likely to be affected by ADHD as girls [4].

There is a high level of comorbidity in children with ADHD with oppositional defiant and conduct disorders [5] as well as anxiety and depressive disorders [6] being the most common comorbid disorders. In addition, children and adolescents with ADHD often struggle with learning disabilities, which affects their academic and educational achievement [7]. Further, ADHD has a significant impact on family functioning [8] and peer relationships [9]. There is also evidence that youths with ADHD are often exposed to stigma and bullying [10]. The broad negative effects of ADHD are often accompanied by a reduced health-related quality of life (HRQoL) in affected children and adolescents, as reported in a recent meta-analysis [11].

HRQoL is considered a multidimensional construct that encompasses physiological, psychological, and social dimensions of an individual’s well-being and functioning [12]. The World Health Organization defines HRQoL as the ‘individual’s perception of their position in life, in the context of culture and value systems in which they live, and in relation to their goals, expectations, standards and concerns’ [13]. In recent years, the concept of HRQoL has become increasingly important when evaluating medical treatments and interventions and is considered an important outcome measure in clinical and epidemiological research [14]. During the COVID-19 pandemic, there was a decline in HRQoL as many areas of children and adolescents’ lives were severely restricted [15]. Considering that children and adolescents with ADHD often have impaired HRQoL, identifying factors that influence their HRQoL can provide important information for the development of prevention and intervention programmes for affected children and adolescents, and thus promote positive mental health and well-being [16]. In public health research, the concept of risk and protective factors has received growing attention in recent years [17]. Risk factors increase the likelihood of negative health outcomes, while protective factors promote positive development and enhance the likelihood of positive health outcomes [18, 19]. Risk and protective factors are usually categorised into personal, familial, and social factors. When considering HRQoL in the context of mental health problems, health care-related factors such as treatment satisfaction may also be important.

Only very few studies have examined the associations of risk and protective factors with HRQoL in children and adolescents with ADHD so far. Most of these studies assessed clinical factors such as symptom severity and comorbidity. In general, children and adolescents with more severe ADHD symptoms and those with multiple comorbid disorders have lower HRQoL [20, 21]. In a longitudinal study among children and adolescents with ADHD, greater internalising mental health problems such as anxiety and depressive symptoms as well as more severe ADHD symptoms at baseline significantly predicted lower HRQoL at 10-year follow-up [22]. Furthermore, some studies have found that the HRQoL of children and adolescents with ADHD improves with effective treatment [23-25]. With regard to familial and social factors, children and adolescents with ADHD who have a parent with a health or mental health problem and those with peer relationship problems have lower HRQoL [21]. In general, parental psychopathology is an important risk factor for child development [26]. Maternal smoking during pregnancy and not living with both parents were also linked to lower HRQoL [21]. Moreover, deficits in emotional stress regulation were also associated with lower HRQoL in children and adolescents with ADHD [25, 27]. Apart from the clinical and psychosocial risk and protective factors mentioned above, further studies reported that older age was associated with lower HRQoL in children and adolescents with ADHD [28].

Despite single study results on HRQoL and on risk and protective factors in children and adolescents with ADHD, there is an overall scarcity of studies on this topic. In particular, there are few largescale population-based studies on health care-related and psychosocial factors affecting HRQoL in this group. Therefore, the current study aims to investigate parent-reported dimensions of HRQoL in a sample of children and adolescents with an administrative ADHD diagnosis and relate them to health care-related factors and psychosocial risk and protective factors. Based on the above-mentioned findings, we expect that psychosocial risk factors such as parental burden and parental psychopathology are associated with lower HRQoL, whereas a positive family climate and a high level of social support are protective factors that are linked to higher HRQoL in children and adolescents with ADHD. Further, we assume that health care-related factors such as psychological health care utilisation are related to higher HRQoL.

## 2. Methods

### 2.1 Study design and conduct

The consortium project INTEGRATE-ADHD was designed as a cross-sectional survey of parents of children and adolescents who had an administratively documented ADHD diagnosis (ICD-10 F90.0-9, Disturbance of activity and attention) in at least one quarter of 2020 (so-called M1Q criterion). Parents were included whose children were insured with the third-largest nationwide operating German statutory health insurance company, DAK-Gesundheit, in the insurance year 2020 and who were 0 to 17 years old at the time and whose administrative ADHD diagnosis was labelled as confirmed with the additional designation ‘G’. The survey was conducted online between October 2021 and August 2022 using modified questionnaires from the epidemiological German Health Interview and Examination Survey for Children and Adolescents (KiGGS study) [29-31] and its in-depth module on mental health (BELLA study) [32, 33]. A subsample of the children and adolescents was additionally examined using guideline-based clinical diagnostics according to the S3 guideline of the ‘Arbeitsgemeinschaft der Wissenschaftlichen Medizinischen Fachgesellschaften’ (AWMF) on ADHD [34].

Of a total of 24,880 invited parents (gross sample), 5,919 parents took part in the online survey. Subsequently, 458 participants were excluded for formal and substantial reasons, such as having more than 50 % missing data or inconsistencies pertaining to information on age and gender between the administrative and the epidemiological data set. This resulted in a net sample of 5,461 participants. The response rate according to AAPOR’s Standard Definitions, Version 9 (RR3) was 21.5 % [35]. For details on sampling and response, see Beyer et al. [36].

The children and adolescents with an administrative ADHD diagnosis who are insured with DAK-Gesundheit can be regarded as approximately representative of the population of all children and adolescents with an administrative ADHD diagnosis insured with statutory health insurance in Germany. A comparison with nationwide outpatient ADHD diagnostics data from the Central Research Institute of Ambulatory Health Care in Germany (Zi) from 2015 and 2016 [37] showed only slight deviations with regard to gender. Younger children, however, were significantly overrepresented and older children and adolescents significantly underrepresented in the INTEGRATE-ADHD gross sample [37]. In order to adjust for deviations of the net sample from the gross sample, population weights were calculated that normalise the net sample to the gross sample, see [36]. The population weights are determined by the inverse probability that a person will participate in the study. People with a low probability of participation represent more people from the population than people with a high probability of participation. The subsequent analyses were performed with weighting.

In the present study, we analysed data from the nationwide epidemiological online survey. Participants with an existing administrative ADHD diagnosis in 2020 could be included in the analyses if they were between 7 and 17 years old and thus had available data on risk and protective factors and if they were currently enrolled in school. The final sample under analysis included *n* = 4,809 parents of children and adolescents aged 7 to 17 years.

**ADHD in Germany – Comparison and integration of administrative and epidemiological ADHD diagnostic data through clinical assessment (INTEGRATE-ADHD)**

**Consortium partners:** Robert Koch Institute Berlin, Department of Epidemiology and Health Monitoring, Germany; University Hospital Würzburg, Department of Child and Adolescent Psychiatry, Psychosomatics and Psychotherapy, Germany; University Medical Centre Hamburg-Eppendorf, Department of Child and Adolescent Psychiatry, Psychotherapy and Psychosomatics, Research Section ‘Child Public Health’, Germany; Vandage GmbH, Germany; University of Würzburg, Germany, Institute for Clinical Epidemiology and Biometry, Germany; DAK-Gesundheit, Germany

**Data holder:** Robert Koch Institute

**Objectives:** Identification of potential causes for the discrepancies between administrative ADHD diagnostic data (based on health insurance claims data) and epidemiological ADHD diagnostic data (based on surveys) for Germany, integration and validation of these data through a guideline-based clinical examination

**Study design:** Cross-sectional online survey, additional clinical examination of a sub-sample, data linkage with administrative health insurance data

**Population:** Children and adolescents who were insured with DAK-Gesundheit in 2020 and who were 0 to 17 years old at that time and for whom an administrative ADHD diagnosis labelled as confirmed was available in at least one quarter

**Gross sample:** 24,880 children and adolescents insured with DAK-Gesundheit with an administrative ADHD diagnosis

**Net sample:** 5,461 surveyed parents, 202 clinically examined children and adolescents

**Data collection period:** October 2021 to August 2022 (online survey), January 2022 to January 2023 (online clinical examination)

More information in German at www.rki.de/integrate-adhd

**Infobox 1**

**Administrative data**

Administrative data is generated as part of administrative procedures. Important data sources for health reporting purposes are the claims data of statutory health insurance funds, from which, for example, prevalence rates (frequencies) of billed medical or psychological diagnoses can be determined. In addition, this data includes information on age and gender of the insured persons, on the utilisation of various outpatient and inpatient healthcare services, drug prescription data and information on the direct costs of utilisation. The administrative diagnostic data on ADHD of children and adolescents used in the project INTEGRATE-ADHD relates to the year 2020 and stems from the statutory health insurance provider DAK-Gesundheit.

**Epidemiological data**

Epidemiological data is collected through surveys and examinations with the aim of researching the prevalence and causes of diseases in the population. Frequencies of diagnosed physical diseases and mental disorders are often assessed by asking the participants whether a doctor (or a psychologist) had diagnosed the respective disease/disorder. The diagnostic data on ADHD in children and adolescents collected in the online survey of the project INTEGRATE-ADHD is based on the parents’ report of an ADHD diagnosis for their child ever made by a medical doctor or psychologist. In addition, the epidemiological data collected in the project INTEGRATE-ADHD also includes questions on sociodemographics (e.g. age and gender of the child, parental education, history of migration), psychopathology and comorbidity (e.g. ADHD symptom severity, ADHD diagnosis of the parents, anxiety, depression), risk and protective factors, quality of life, as well as satisfaction with care and barriers to utilisation.

### 2.2 Instruments

#### Health-related quality of life (HRQoL)

HRQoL was assessed using the parent-reported KIDSCREEN-27 [38]. The KIDSCREEN-27 is composed of five dimensions including: Physical Well-Being (five items), Psychological Well-Being (seven items), Autonomy and Parent Relations (seven items), Peers and Social Support (four items), and School Environment (four items). Responses indicate intensity or frequency and are based on a 5-point Likert scale ranging from ‘never’ (1) to ‘always’ (5), wherein higher scores suggest better HRQoL. The scores of the five scales of the KIDSCREEN-27 can be converted into T-values according to the manual, which result in the following categories: T-values below 40 indicate low HRQoL, T-values between 40 and 60 indicate medium HRQoL, and T-values above 60 indicate high HRQoL. Please note that no HRQoL total score was calculated, but each dimension of the KIDSCREEN-27 was analysed separately.

#### Psychological health care utilisation due to ADHD

Information regarding utilisation of health care services due to ADHD was collected using items from the KiGGS and BELLA studies. Utilisation of health care services was operationalised with one dichotomous variable asking if the child or adolescent was currently receiving psychological, psychotherapeutic or psychiatric treatment for ADHD. This question was only presented to those parents who had previously indicated that their child had an ADHD diagnosis. A filter-related missing in this variable (because the child did not have a parent-reported ADHD diagnosis) was considered as no psychological health care utilisation in the analyses.

#### Psychosocial risk and protective factors

Parental psychopathology was measured with the short version of the multidimensional Symptom-Checklist 90-R (SCL-K-9) [39]. The nine items result in a sum score ranging from 0 to 36, with higher means indicating increased psychopathology. Parental burden was assessed with a well-established 11-item scale on parental burden from the BELLA study [33]. The sum score ranged from 0 to 13, with higher means indicating higher burden. Familial protective factors were captured by using a shortened version of the Family Climate Scale [40] that was also implemented in the KiGGS and BELLA studies [41]. The nine items (e.g., ‘in our family, everyone is responsive to each other’s concerns and needs’) can be answered on a 4-point scale ranging from ‘not true’ (1) to ‘exactly true’ (4). Social resources were evaluated by means of the German translation of the Social Support Scale [42]. The eight items of this scale measure how often support is experienced in the form of listening, affection, help with problem solving, and the opportunity to do things together. The 5-point response categories ranged from ‘never’ (1) to ‘always’ (5). The sum scores of the Family Climate Scale and the Social Support Scale were transformed into values between 0 and 100, with higher values indicating higher levels of the protective factors.

#### Sociodemographics

Age in years, gender, parental education (1 = low, 2 = medium, 3 = high education according to the Comparative Analysis of Social Mobility in Industrial Nations (CASMIN) classification), and migration background (1 = no, 2 = yes) were assessed as sociodemographic indicators. The administrative data contains binary information on gender. The gender of 27 children was reported as ‘diverse’ in the online survey. As this group was too small to be statistically analysed, these individuals were assigned the gender information from the administrative data in the course of the data linkage. Also, if no information on gender was provided in the survey, the information was taken from the administrative data (*n* = 2). The gender was therefore included in the analyses with the characteristics girl/female and boy/male.

#### ADHD symptom severity

To assess the degree of impairment due to ADHD symptoms, the parent-reported German ADHD Rating Scale (FBB-ADHS) [43] was used. The scale is composed of 20 items that correspond to ADHD symptom criteria of the ICD-10 and DSM-5. Answers reflect the severity of ADHD symptoms and range from ‘not true’ (0) to ‘especially true’ (3). Erhart et al. [44] found that the FBB-ADHS demonstrated reliability and good to excellent internal consistency (Cronbach’s α = 0.73 – 0.90) as well as factorial validity (root mean square error of approximation, RMSEA = 0.06).

### 2.3 Statistical analyses

Descriptive analyses were performed including the calculation of frequencies, means and standard deviations for analysed variables. Further, the prevalences of low, medium and high HRQoL according to the five dimensions of the KIDSCREEN-27 were calculated for girls and boys. Correlations between the predictor variables were calculated using Pearson correlations, point-biserial correlations, and Spearman correlations. Multivariate analyses using linear regression models were used to assess how health care-related and psychosocial risk and protective factors (i.e. psychological health care utilisation, parental burden, parental psychopathology, family climate, and social support) are associated with HRQoL in children and adolescents with ADHD. Five regression models were run, wherein each dimension of the KIDSCREEN-27, i.e. Physical Well-Being, Psychological Well-Being, Autonomy and Parent Relations, Peers and Social Support, and School Environment, served as an outcome. Age, gender, parental education, migration background, and ADHD symptom severity served as control variables in all five regression models. Significant effects were assumed at a significance level of *p* < 0.05. All analyses were performed using IBM SPSS version 27.

## 3. Results

### 3.1 Descriptive statistics

Data from parent proxy-reports of *n* = 4,809 families with children currently enrolled in school between the ages of 7 to 17 years (*M*_age_ = 12.30, *SD*_age_ = 2.67, 25.1 % female) were analysed. Most of the children and adolescents had no migration background (*n* = 4,370, 90.9 %) and the majority of their parents had a medium level of education (*n* = 2,889, 60.1 %). The mean HRQoL of the participants ranged between 44.8 (Physical Well-Being) and 52.8 (Autonomy and Parent Relations). Further details on the sociodemographic and psychosocial characteristics of the analysed sample are presented in Table 1.

### 3.2 HRQoL of children and adolescents with ADHD

**Figure 1:**
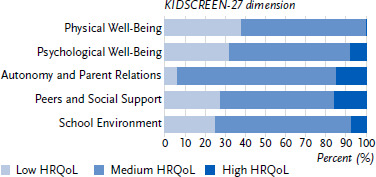
HRQoL of girls with ADHD according to the five dimensions of the KIDSCREEN-27 (Physical Well-Being: *n* =1,190; Psychological Well-Being: *n* =1,187; Autonomy and Parent Relations: *n* =1,185; Peers and Social Support: *n* =1,189; School Environment: *n* =1,188). Source: INTEGRATE-ADHD, online survey

**Figure 2:**
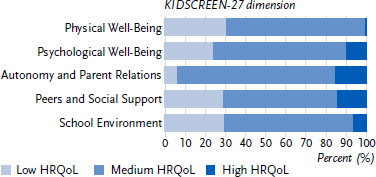
HRQoL of boys with ADHD according to the five dimensions of the KIDSCREEN-27 (Physical Well-Being: *n* = 3,539; Psychological Well-Being: *n* = 3,524; Autonomy and Parent Relations: *n* = 3,532; Peers and Social Support: *n* = 3,533; School Environment: *n* = 3,522). Source: INTEGRATE-ADHD, online survey

Normal HRQoL was reported by 56.8 % to 79.3 % of the girls and 56.8 % to 78.3 % of the boys with an administrative ADHD diagnosis for all five dimensions of HRQoL. The largest proportion of low HRQoL was reported for the dimensions Physical Well-Being (37.5 %), Psychological Well-Being (31.8 %) and Peers and Social Support (27.2 %) for girls, and for the dimensions Physical Well-Being (30.0 %), School Environment (28.9 %) and Peers and Social Support (28.4 %) for boys. The highest levels of high HRQoL were reported for the dimensions Autonomy and Parent Relations (girls: 14.9 %; boys: 16.1 %) and Peers and Social Support (girls: 16.1 %; boys: 14.8 %). See Figure 1 and Figure 2 for full details.

### 3.3 Correlations among predictor variables

The correlations between the predictor variables are presented in Table 2. The sociodemographic variables were only weakly correlated with each other and with the risk and protective factors, with correlation coefficients up to *r* = 0.20. Correlations between the risk and protective factors were also found to be weak. The highest intercorrelation was found between ADHD symptom severity and parental psychopathology (*r* = 0.33).

### 3.4 Risk and protective factors for HRQoL

The results of the multivariate linear regression analyses on risk and protective factors for the five dimensions of HRQoL are reported in Table 3 and described below.

#### Physical Well-Being

Being female, being older, parental burden, parental psychopathology, and currently receiving psychological treatment for ADHD were associated with lower physical well-being in children and adolescents with ADHD. Meanwhile, better family climate and more social support were associated with higher physical well-being, whereby family climate was most strongly associated with physical well-being (ß = 0.20, *p* < 0.001).

#### Psychological Well-Being

Being female, being older, showing more severe ADHD symptoms, parental burden, parental psychopathology, and currently receiving psychological treatment for ADHD were associated with lower psychological well-being in children and adolescents with ADHD, with ADHD symptom severity being the strongest risk factor (ß = -0.23, *p* < 0.001). However, having a better family climate and more social support were associated with higher psychological well-being, with social support being more strongly associated with psychological well-being (ß = 0.26, *p* < 0.001).

#### Autonomy and Parent Relations

Being female, showing more severe ADHD symptoms, increased parental burden, and parental psychopathology were found to be associated with lower scores on autonomy and parent relations in children and adolescents with ADHD. Meanwhile, being older, having parents with medium or high education, having a better family climate, and social support were associated with higher scores on autonomy and parent relations, with social support being the strongest protective factor (ß = 0.43, *p* < 0.001).

#### Peers and Social Support

More severe ADHD symptoms, increased parental burden, and currently receiving psychological treatment for ADHD were associated with lower scores on peers and social support. However, being older, having better family climate, and increased social support were associated with higher scores on peers and social support among children and adolescents with ADHD, with social support being the strongest protective factor (ß = 0.21, *p* < 0.001).

#### School Environment

Older age, more severe ADHD symptoms and increased parental burden were associated with lower scores on the dimension School Environment, with ADHD symptom severity being the strongest risk factor (ß = -0.41, *p* < 0.001). Conversely, better family climate and increased social support were associated with higher scores, whereby social support was the stronger protective factor (ß = 0.20, *p* < 0.001).

## 4. Discussion

The aim of the present study was to identify risk and protective factors associated with dimensions of HRQoL in a sample of children and adolescents with an administrative ADHD diagnosis. As expected, we found that parental burden and parental psychopathology were associated with lower scores on most dimensions of HRQoL, indicating that these are risk factors for lower HRQoL in children and adolescents with ADHD. With regard to protective factors, we found that having a better family climate and more social support were associated with higher HRQoL in children and adolescents with ADHD. Contrary to our hypothesis, psychological health care utilisation was associated with lower HRQoL. Moreover, we found some individual effects for ADHD symptoms severity and the sociodemographic variables age, gender, and parental education on some dimensions of HRQoL.

Findings of our descriptive analysis indicated that around a quarter to a third of children and adolescents with ADHD reported low HRQoL in terms of the dimensions Physical Well-Being, Peers and Social Support, Psychological Well-Being, and School Environment, with Physical Well-Being being the most impaired in both girls and boys. In addition, girls with ADHD primarily showed impairments in their Psychological Well-Being, while boys were more affected in terms of the dimensions School Environment and Peers and Social Support. The mean HRQoL of the participants in the present study was slightly below the mean value of the European norm sample (T-value < 50) in almost all dimensions with T-values between 44.75 (Physical Well-Being) and 47.82 (Peers and Social Support). Only the T-value for the Autonomy and Parent Relations dimension was slightly above European average at 52.84. Compared to a German population-based sample of children and adolescents aged 11 to 17 years of KiGGS Wave 2 (2014 – 2017) [45], the children and adolescents with an administrative ADHD diagnosis in the present study had slightly lower T-values across all HRQoL dimensions (Physical Well-Being: 44.75 vs. 48.95, Psychological Well-Being: 46.75 vs. 50.67, Autonomy and Parent Relations: 52.84 vs. 54.14, Peers and Social Support: 47.82 vs. 51.16, School Environment: 45.89 vs. 51.29). However, it should be noted that the COVID-19 pandemic has also led to a decline in HRQoL among children and adolescents [15], which makes comparison with data from KiGGS Wave 2 difficult.

In terms of psychosocial risk factors, parental burden and parental psychopathology were associated with lower scores on most dimensions of HRQoL. This is consistent with previous research [20] and emphasises the importance of considering parental mental health in targeted interventions for children and adolescents with ADHD. As parental psychopathology is an important risk factor for child development [26], parental psychopathology (including subclinical symptoms) and the associated parent-child interaction should be considered more strongly in child psychiatric diagnosis and treatment. Further, children and adolescents currently receiving psychological treatment for ADHD reported lower scores on the dimensions Physical Well-Being, Psychological Well-Being and Peers and Social Support, which was contrary to our hypothesis. This could be explained by the fact that children and adolescents who are currently undergoing medical treatment because of their ADHD may be particularly burdened and have a high level of psychological stress, which negatively impacts their HRQoL. This is in line with research showing that children and adolescents with more severe ADHD symptoms have lower HRQoL [20, 21]. It can be expected that with advanced or completed effective treatment, psychological stress will decrease and thus HRQoL will improve, as previous studies have indicated [23-25].

Regarding the examined psychosocial protective factors, we found beneficial effects of a positive family climate and social support on HRQoL. Children and adolescents with ADHD who had a positive family climate and a high level of social support reported higher scores on all dimensions of HRQoL, with social support being the strongest protective factor for all HRQoL dimensions except Physical Well-Being. The results highlight the importance of prevention and intervention programmes for children and adolescents with ADHD that aim to support the relationship between parents and their children and to strengthen resources such as the availability of good social support. Such programmes may aid children and adolescents in coping with the wide-ranging negative effects of ADHD on school, family functioning, and peer relationships that have been reported in the literature [7-9]. Children and adolescents with ADHD may particularly benefit from cognitive-behavioural therapies (CBT) with a focus on improving social skills to promote positive peer relationships and friendships. There is extensive scientific evidence of the effectiveness of CBT for children and adolescents with ADHD [46, 47]. In addition, peer inclusion interventions have been reported to improve the social functioning of children and adolescents with ADHD [48] which may have a positive impact on their HRQoL.

In terms of sociodemographic variables, we found that girls had a higher risk for low HRQoL regarding their physical well-being, psychological well-being, autonomy, and parent relations compared to boys. The fact that girls have lower HRQoL than boys has already been confirmed in other studies – irrespective of the presence of an ADHD diagnosis [45, 49]. Further, mixed results were found for age. Whereas being older was associated with lower HRQoL in terms of physical well-being, psychological well-being, and school environment, older adolescents also displayed higher scores on autonomy and parent relations as well as peers and social support. These results underline the importance of age- and gender-sensitive prevention and intervention programmes for children and adolescents with ADHD that meet their specific needs and could be implemented in school or community settings.

The present study has the following limitations: By means of the variables included in our regression models, a proportion of 15 % to 37 % of the variance in HRQoL was explained. This may indicate that HRQoL in children and adolescents with ADHD is associated with other important factors that we did not consider in our model. Further, the effects of the identified risk and protective factors on HRQoL were mostly small. Our investigation did not assess the impact of ADHD medications on ADHD symptoms or HRQoL as that was beyond the scope of these analyses. Future studies may take this into account. In addition, there are some overlaps in the predictors and dimensions of HRQoL in terms of their content, which primarily concerns the predictors family climate and social support as well as the dimensions Autonomy and Parent Relations and Peers and Social Support. This should be considered when interpreting the results, and it is thus not surprising that we found protective effects of family climate and social support on these dimensions of HRQoL. Further, it should be noted that INTEGRATE-ADHD is a cross-sectional observational study that only identifies associations between risk and protective factors and HRQoL in a sample of children and adolescents with an administrative ADHD diagnosis and no cause-effect relationships. Moreover, the sample under analysis only included children and adolescents aged 7 to 17 years as the analysed risk and protective factors are only intended for use in this age group. Considering that around 25 % of affected children are first diagnosed with ADHD before the age of six [50], future analyses may also include younger children. Lastly, HRQoL in children and adolescents and risk and protective factors were assessed by parent-reports. A systematic review found that there is a difference between child self-ratings and parent proxy-ratings in the assessment of HRQoL in children with ADHD, in that children rate their HRQoL better than their parents [51].

The strengths of the present study include the large sample size, the assessment of risk and protective factors using established measures, and that, after weighting, the study data can be considered approximately representative of children and adolescents with an administrative ADHD diagnosis in Germany. However, the weighting does not adjust for the specific ‘not missing at random’ non-response of parents of children with ADHD who either did not participate because they were certain that their child had ADHD or those who did not participate because they were certain that their child did not have ADHD. Only 6.1% of the participants indicated a migration background for their child with ADHD, which is considerably lower than the proportion of 34% in the German population [52]. However, ADHD might be considerably underdiagnosed in youths with a migration background [53]. In the KiGGS study, for example, the proportion of youths with a parent-reported ADHD diagnosis from families with a two-sided migration background (mother and father) was only 1.4%, while the proportion of youths with ADHD from families without a migration background was 5.1%. Further, the proportion of youths with clinically relevant ADHD symptoms but without a diagnosis was 11.0% in migrant families and 7.1% in non-migrant families [54].

To the best of our knowledge, this is one of the first studies to investigate health care-related and psychosocial factors affecting dimensions of HRQoL in children and adolescents with an administrative ADHD diagnosis. The findings emphasise the importance of parental psychopathology as a risk factor and family climate and social support as protective factors for HRQoL of children with ADHD. Given the fact that ADHD in children and adolescents is highly prevalent and causes significant impairments in HRQoL, our findings can inform the development of prevention and intervention programmes for children and adolescents with ADHD and thus positively influence their mental health and well-being.

## Key statements

- A quarter to a third of children and adolescents with ADHD reported low HRQoL across the five dimensions.
- Parental psychopathology and parental burden prove to be risk factors for lower HRQoL in children and adolescents with ADHD.
- A good family climate and social support prove to be protective factors and are associated with higher HRQoL in children and adolescents with ADHD.
- Findings highlight the importance of considering parental mental health in targeted interventions for children and adolescents with ADHD.
- Identifying factors that affect HRQoL can inform the development of prevention and intervention programmes for children and adolescents with ADHD.

## Figures and Tables

**Table 1:** Description of the analysed sample of children and adolescents. Source: INTEGRATE-ADHD, online survey

	INTEGRATE-ADHD Sample	
	*n* (%)	*M (SD)*
Age (7 to 17 years)		12.30 (2.67)
**Gender**		
Female	1,208 (25.1)	
Male	3,601 (74.9)	
**Parental education**		
Low	499 (10.4)	
Medium	2,889 (60.1)	
High	1,211 (25.2)	
No information	210 (4.4)	
**Migration background**		
No	4,370 (90.9)	
Yes	292 (6.1)	
No information	147 (3.1)	
**HRQoL**		
Physical Well-Being		44.8 (9.09)
Psychological Well-Being		46.8 (11.49)
Autonomy and Parent Relations		52.8 (9.52)
Peers and Social Support		47.8 (14.23)
School Environment		45.9 (10.77)
ADHD symptom severity		1.3 (0.68)
**Psychological health care utilisation**		
Yes	1,963 (40.8)	
No	2,846 (59.2)	
Parental burden		9.13 (3.33)
Parental psychopathology		8.58 (6.77)
Family climate		61.26 (16.54)
Social support		82.11 (15.59)

*M* = mean, *SD* = standard deviation, CASMIN = Comparative Analysis of Social Mobility in Industrial Nations, HRQoL = health-related quality of life, ADHD = attention-deficit/hyperactivity disorder

**Table 2:** Zero-order correlations among predictor variables. Source: INTEGRATE-ADHD, online survey

Variables	1.	2.	3.	4.	5.	6.	7.	8.	9.	10.
1. Gender^a^	-									
2. Age^b^	0.02	-								
3. Parental education^c^	-0.01	-0.01	-							
4. Migration background^a^	0.01	-0.02	-0.01	-						
5. ADHD symptom severity^b^	-0.08^**^	-0.20^**^	-0.04^**^	0.00	-					
6. Psychological health care utilisation^a^	-0.01	0.15^**^	-0.03	-0.03	-0.19^**^	-				
7. Parental burden^b^	-0.01	-0.07^**^	-0.00	-0.01	0.24^**^	-0.06^**^	-			
8. Parental psychopathology^b^	0.01	-0.02	-0.05^**^	-0.02	0.33^**^	-0.06^**^	-0.19^**^	-		
9. Family climate^b^	0.01	-0.20^**^	-0.05^**^	0.05^**^	-0.14^**^	-0.04^*^	0.17^**^	-0.20^**^	-	
10. Social support^b^	0.05^**^	-0.20^**^	-0.01	0.08^**^	-0.11 ^**^	-0.02	0.18^**^	-0.14^**^	-0.14^**^	-

Zero-order correlations are presented by Pearson correlations for continuous variables, point-biserial correlations for one dichotomous and one continuous variable or for two dichotomous variables, and Spearman correlations for one ordinal and dichotomous or continuous variable.

^a^dichotomous variable,

^b^continuous variable,

^c^ordinal variable,

^*^p ≤ 0.05,

^**^p ≤ 0.01,

^***^p ≤ 0.001

ADHD = attention-deficit/hyperactivity disorder

**Table 3:** Results of the multivariate linear regression analyses on factors predicting HRQoL in children and adolescents with ADHD. Source: INTEGRATE-ADHD, online survey

	KIDSCREEN-27 Dimension																			
	Physical Well-Being (*n* = 4,565)				Psychological Well-Being (*n* = 4,547)				Autonomy and Parent Relations (*n* = 4,522)				Peers and Social Support (*n* = 4,559)				School Environment (*n* = 4,558)			
	*b*	*SE*	ß	*p*	*b*	*SE*	ß	*p*	*b*	*SE*	ß	*p*	*b*	*SE*	ß	*p*	*b*	*SE*	ß	*p*
*Constant*	16.44	0.47		<0.001	24.83	0.58		<0.001	17.52	0.44		<0.001	9.68	0.57		<0.001	15.55	0.43		<0.001
**Control variables**																				
Gender (Ref: male)	**-0.80**	**0.11**	**-0.10**	**<0.001**	**-1.44**	**0.13**	**-0.14**	**<0.001**	**-0.48**	**0.10**	**-0.06**	**<0.001**	-0.05	0.13	-0.01	0.705	-0.01	0.10	-0.00	0.890
Age	**-0.34**	**0.02**	**-0.26**	**<0.001**	**-0.18**	**0.02**	**-0.10**	**<0.001**	**0.17**	**0.02**	**0.12**	**<0.001**	**0.05**	**0.02**	**0.04**	0.014	**-0.16**	**0.02**	**-0.13**	**<0.001**
Parental education (Ref: Low)																				
Medium	-0.27	0.15	-0.04	0.077	-0.01	0.19	-0.00	0.939	**0.43**	**0.14**	**0.06**	**0.003**	0.09	0.18	0.01	0.641	-0.07	0.14	-0.01	0.637
High	-0.15	0.17	-0.02	0.350	-0.16	0.21	-0.02	0.423	**0.41**	**0.16**	**0.05**	**0.009**	0.11	0.20	0.01	0.585	0.00	0.15	0.00	0.976
Migration background	0.25	0.19	0.02	0.194	0.12	0.24	0.00	0.622	-0.15	0.18	-0.10	0.406	0.43	0.23	0.03	0.062	0.38	0.17	0.03	0.029
ADHD symptom severity	-0.11	0.08	-0.02	0.148	**-1.55**	**0.10**	**-0.23**	**<0.001**	**-0.63**	**0.07**	**-0.12**	**<0.001**	**-0.97**	**0.09**	**-0.16**	**<0.001**	**-1.93**	**0.07**	**-0.41**	**<0.001**
**Risk factors**																				
Parental burden	**-0.05**	**0.02**	**-0.04**	**0.004**	**-0.06**	**0.02**	**-0.04**	**0.003**	**-0.06**	**0.02**	**-0.05**	**<0.001**	**-0.05**	**0.02**	**-0.04**	**0.008**	**-0.04**	**0.01**	**-0.04**	**0.003**
Parental psychopathology	**-0.03**	**0.01**	**-0.06**	**<0.001**	**-0.12**	**0.01**	**-0.17**	**<0.001**	**-0.04**	**0.01**	**-0.07**	**<0.001**	-0.02	0.01	-0.03	0.067	-0.01	0.01	-0.22	0.135
**Protective factors**																				
Psychological health care utilisation	**-0.22**	**0.10**	**-0.03**	**0.026**	**-0.40**	**0.12**	**-0.04**	**0.001**	0.05	0.09	0.01	0.607	**-0.59**	**0.12**	**-0.07**	**<0.001**	0.15	0.09	0.02	0.097
Family climate	**0.04**	**0.00**	**0.20**	**<0.001**	**0.07**	**0.00**	**0.13**	**<0.001**	**0.04**	**0.00**	**0.17**	**<0.001**	**0.02**	**0.00**	**0.10**	**<0.001**	**0.01**	**0.00**	**0.06**	**<0.001**
Social support	**0.03**	**0.00**	**0.13**	**<0.001**	**0.08**	**0.00**	**0.26**	**<0.001**	**0.10**	**0.00**	**0.43**	**<0.001**	**0.06**	**0.00**	**0.21**	**<0.001**	**0.03**	**0.00**	**0.16**	**<0.001**
Model fit	*F*[11,4553] =114.67				*F*[11,4535]=198.44				*F*[11,4540] = 242.85				*F*[11,4547] = 71.56				*F*[11,4546] =135.06			
	*p* < 0. 001				*p* < 0. 001				*p* < 0. 001				*p* < 0. 001				*p* < 0. 001			
	*adjusted R^2^* = 0.215				*adjusted R^2^* = 0.323				*adjusted R^2^* = 0.369				*adjusted R^2^* = 0.146				*adjusted R^2^* = 0.244			

Weighted data, Ref. = reference category, *SE* = standard error, HRQoL = health-related quality of life, ADHD = attention-deficit/hyperactivity disorder
